# Cultural diversity in urban park use and perception

**DOI:** 10.1007/s44327-026-00247-7

**Published:** 2026-04-29

**Authors:** Tural Aliyev, Manuel Fischer, Janine Bolliger, Katrin Pakizer, Natascha Zinn, Noah Schmid

**Affiliations:** 1 Environmental Social Sciences Department, Swiss Federal Institute of Aquatic Science and Technology, Eawag, Switzerland; 2 Forest Health ad Biotic Interactions Forest Entomology Unit, Swiss Federal Institute for Forest, Snow and Landscape Research, Birmensdorf, Switzerland

**Keywords:** Cultural background, Cultural ecosystem services, Cultural diversity, Urban society, Nature perception, Urban green areas

## Abstract

Given increasing migration and cultural diversification in urban contexts, understanding how people with different cultural backgrounds use and perceive urban green areas is relevant for inclusive planning. This exploratory, context-specific study examines how park users categorized as Swiss and non-Swiss report motivations, emotions, and ecological preferences in four public parks in Zurich, Switzerland. Based on 100 face-to-face go-along interviews, the study applies a mixed-methods design combining descriptive quantitative summaries with qualitative insights. Findings show shared appreciation for health-related benefits across both groups, alongside patterns of variation in reported motivations, emotional experiences, and preferences for vegetation structure. These patterns are discussed descriptively in relation to a simple Swiss vs. non-Swiss cultural background. The article contributes to urban studies by applying a three-dimensional framework—motivational, emotional, and ecological—that considers vegetation characteristics alongside reported user experiences, offering context-specific insights into culturally differentiated park use in Zurich.

## Introduction

Given the current rate of migration and globalization, ensuring inclusivity regarding the expectations and needs of people with different cultural backgrounds living together in urban environments is important [46, 47]. Especially, urban green areas (UGAs) such as public parks, which fulfil crucial services for human well-being, should be designed to be attractive and therefore used by all people [27]. Gaining specific insights into the use and perception of users of UGAs and considering their diverse needs and preferences can therefore enhance the design and management of UGAs, and make access to it more equitable [4, 23, 48]. This article analyzes how the cultural background of park users influences their use and perception of parks through three key dimensions. Motivational, emotional, and ecological dimensions are conceptualized as jointly contributing to the understanding of how cultural backgrounds relate to individuals’ use and perceptions of urban green areas (UGAs). This integrated approach through three dimensions allows for a comprehensive analysis of the interplay between culture and environment in shaping human experiences of parks [11, 48].

Few studies exist on the perceptions, preferences, and utilization patterns of parks based on cultural background in European and Swiss cities, as compared to the well-established tradition in North America [14, 19, 20, 41, 42]. This article addresses this gap by examining the use of UGAs in Zurich, Switzerland. The study was conducted across four urban parks in the city, which provides ideal research setting with its diverse population of 32% non-Swiss residents as of 2020 [24]. Cultural background is operationalized as Swiss or non-Swiss, based on information about place of birth and primary language. This simple dichotomy assumes that longer-term socialization processes regarding the use and perception of UGA differ between Swiss and non-Swiss. While both groups are obviously internally heterogeneous (e.g., non-Swiss include migrants as well as tourists), this simplified dichotomy still provides first insights into how cultural background matters. Further, the internal heterogeneity of both groups is considered by drawing not only on quantitative but also qualitative evidence derived from 100 semi-structured interviews.

Addressing the research question of how cultural background of park users influences their use and perception of parks is important for several reasons and provides related contributions to the literature. Firstly, relating ecological factors such as vegetation structure to the study of park use in the context of cultural background [12, 19, 26, 38], this study contributes to the research in urban studies. The integration of different perspectives and our proposal of analyzing them along three dimensions provides a holistic understanding of how urban parks function across diverse cultural contexts and how users perceive ecological park structures. Secondly, this research offers a thorough analysis of the cultural backgrounds of park users, going beyond going beyond traditional demographic factors such as age and gender [11, 20], by incorporating the country of origin and comparing Swiss and non-Swiss users. Applying an interdisciplinary approach combining sociological and ecological factors has become central to understanding social behaviors toward natural elements in recent years [18]. Such an approach enriches the understanding of the complex relationships between cultural backgrounds, ecosystem services, and the perception of ecological structures within urban parks. It highlights how cultural diversity influences the use and perception of ecosystem services provided by UGAs, offering qualitative descriptions and explanations that are complemented with quantitative data. Third, in a context of migration and globalization, considering the cultural background of users has become an imperative in the development of UGAs [42]. This is crucial to ensure inclusivity in cities and to cater to the diverse expectations and needs of urban society, thereby responding to calls for environmental justice, and greater participation of different parts of the population [41].

## Literature review and conceptual framework

### Use and perceptions of urban green areas

Traditionally, UGAs encompass diverse components such as parks, street trees, urban agriculture, residential lawns, and roof gardens [9]. UGAs play a critical role for various ecosystem services such as climate adaptation, biodiversity support, and the incorporation of individual preferences and needs of urban residents, yet the interactions among them remain underexplored [31]. UGAs provide numerous social benefits for urban dwellers, including enhancements in mental and physical well-being through stress reduction and opportunities for relaxation [28, 30, 33]. Additionally, UGAs improve quality of life by offering both active and passive recreational opportunities, such as engaging in physical activities like sports, playing with children, or walking pets.

Passive activities encompass activities such as unwinding, painting, sunbathing, socializing, interacting with others, and simply immersing oneself in nature [11]. Moreover, UGAs play a vital role in enhancing the perception of safety [30] and serve as gathering places for residents, thereby promoting social interaction [35]. Recent systematic reviews highlight the growing importance of data-driven methods in advancing the understanding of human–environment interactions in urban contexts [16]. Specifically, they demonstrate how the integration of spatial, ecological, and socio-behavioral datasets enables more fine-grained analyses of UGAs use. However, negative effects on the perception of urban residents regarding UGAs have also been observed. Some surveys have indicated that individuals may feel insecure and fearful of crime in densely vegetated, unmanaged areas with limited visibility [8, 40]. In highly urbanized regions, enclosed UGAs are associated with reduced feelings of social safety, whereas the opposite is true in more rural areas [34]. Urban expansion often reduces access to green spaces, as seen in the case of Damascus, where residential growth has significantly limited everyday contact with nature [22]. Accessibility of green spaces is not only a question of daily leisure but also of safety and resilience, as illustrated by studies on their role as assembly areas in disaster contexts such as Kastamonu, Turkey [25]. This emphasizes that individuals’ motivations for park use and the emotions they have when using parks are linked to ecological aspects of how UGAs are designed. To address the variety of potential motivations and perceived benefits, a three-dimensional framework of park use and preferences is introduced, distinguishing between a motivational dimension (why do individuals come to parks and what do they do in parks?), an emotional dimension (how satisfied are individuals with parks and how do they feel?), and an ecological dimension (what is individual’s preference for nature in parks?). The literature discussed below hints towards the importance of all three dimensions.

### Cultural background

The cultural background of users can influence their perceptions, preferences, and utilization of UGAs [12, 19, 26, 38]. The term “cultural origin” or “cultural background” pertains to the ethnic or cultural heritage inherited from one’s ancestors [44]. Ethnicity is a concept that is multidimensional and adaptable, generally described as rooted in a collective comprehension of a group’s historical and territorial roots (both regional and national), along with specific cultural traits like language and/or religion [47]. Beyond ethnicity, cultural backgrounds encompass factors like nationality, religion, language, and traditions [47]. All these factors can shape people’s values, beliefs, and behaviors, which, in turn, may impact their interactions with UGAs in different ways.

First, different cultural groups may have specific practices and traditions associated with nature and green spaces, such as picnicking, socializing, or engaging in outdoor activities in parks or gardens [26, 27, 38]. These traditions associated with specific cultural backgrounds can shape how individuals perceive and utilize UGAs. Second, cultural backgrounds can influence social norms and behaviors within UGAs. For instance, some cultures may particularly value family gatherings or communal interactions in outdoor spaces [26, 38]. Users with such a cultural background may prioritize UGAs that provide suitable facilities for group activities or cultural events. Third, cultural backgrounds can also shape recreational preferences [11]. Some cultures may have a strong inclination towards specific activities such as jogging, yoga, tai chi, or meditation in outdoor settings. Consequently, individuals from these cultural backgrounds might gravitate towards UGAs that can accommodate their preferred recreational activity. Fourth, some cultural backgrounds have spiritual or religious beliefs that attach significance to nature and green spaces [7]. For example, certain Indigenous cultures maintain profound connections with nature, viewing it as sacred. Incorporating cultural stewardship in urban spaces, guided by Indigenous knowledge, can promote nature restoration while fostering a renewed connection to the environment [49]. Individuals from these cultural backgrounds may seek UGAs for spiritual practices, rituals, or contemplation, highlighting the importance of both motivational and emotional dimensions of understanding park use and preferences.

Previous studies on the impact of cultural background on UGAs use and preferences in the North American context reveal that ethnic minorities tend to prefer well-managed landscapes and show less inclination towards wilderness or naturalistic landscapes [49]. Byrne and Wolch [11] suggest that cultural background and ethnicity can influence the choice of active and passive activities. They find that individuals in the US with different cultural backgrounds exhibit distinct preferences. Regarding the European context, Jay and Schraml [26] discovered that the cultural background influenced the utilization of urban forests in their German case study. They observed variations in recreational preferences among different groups. Turkish study participants placed importance on group activities like barbequing and football, particularly in forested areas at the boundary between the city and woodland. On the other hand, the Balkan and Russian-German participants exhibited a preference for forests located beyond the city-woodland interface. They were often accompanied by fewer friends or family members and engaged in activities such as hiking and fruit picking.

Peters et al. [38] also observed in Netherlands that non-Western immigrants tend to visit urban parks in groups, which can be attributed to their strong emphasis on family values. According to Peters et al. [38] there are differences in the relationship with UGAs between the first generation (born in a non-Western country) and second generation (born in a Western country) of non-Western migrants. Regarding their cultural affiliation, the second generation is usually placed between native Western individuals and the first generation of non-Western migrants, which is why they are typically more familiar with nature areas and urban parks than the first generation. Moreover, Gentin [19] highlights that ethnic minorities in Europe may have more utilitarian or family-oriented perceptions of nature and green space, influenced by cultural background and migration experience, compared to the majority population. This is due to the influence of an individual’s self-construct, meaning how one perceives themselves in relation to others, on their thoughts, emotions, and motivations [36]. People from Western Europe and European-Americans are more likely to have an independent self-construct, placing a higher value on uniqueness and self-expression. In contrast, individuals from Asia, Latin America, Africa, and Southern Europe tend to have an interdependent self-concept [36]. An example that highlights the impact of different types of self-construct is outdoor recreation, as this activity holds greater importance for individuals with an independent self-construct (common among people from Western Europe and North America) than for those with an interdependent self-construct [48]. Ethnicity and culture thus play a significant role in motivating individuals for outdoor recreation [48].

Studies have further shown that in Switzerland, Swiss residents exhibit more skepticism towards the activities offered in urban public green spaces compared to foreigners, according to the responses from inhabitants of Geneva [42]. Recent research in Zurich shows that the frequency of green space visits depends not only on personal and situational factors, but also on ecological attributes such as size and the presence of water bodies, highlighting important design elements for culturally diverse park use [15].

Differing perceptions of UGAs can also lead to conflicts between cultural groups. For instance, a popular picnic spot near Zurich, situated on the edge of a forest, was frequently occupied by people from Serbia, Macedonia, and Turkey, leading to tension with nearby Swiss residents [42]. What may have contributed to tensions is that the population in the urban periphery of Zurich tends to be more homogeneous compared to the city center, where diverse cultural groups intermingle, thus creating greater potential for integration [42]. This example highlights the need for a more equitable use of these spaces [17].

Finally, a study on the Tempelhofer Park in Berlin, Germany found that meeting and communication points, as well as designated areas for barbecuing, are particularly crucial preferences for immigrants and older individuals [27]. The research also revealed that individuals aged 65 and above were underrepresented in the park compared to their proportion in the surrounding areas. This “underuse” by older individuals could be attributed to current park infrastructure limitations, as Tempelhofer Park lacks sufficient tree cover for shade and has a shortage of seating and dining areas.

A more recent studies which compare the users’ needs and preferences between Switzerland and Iran also provide interesting insights [5, 6]. The studies have shown that the interviewees expressed a stronger sense of connection and attachment to historically significant parks, as they were able to associate themselves with these places and form emotional bonds [5, 6]. In contrast, modern green spaces provide fewer opportunities for such connections through memories, meanings, and historical significance [5, 6]. This study further demonstrates that while historically rich park structures may hold little to no significance for bi-cultural migrants, they can evoke memories of cultural origins for others.

Beyond functional and ecosystem service perspectives, urban green areas can also be understood as socio-cultural and identity-forming spaces. Urban parks often serve as arenas where belonging, cultural expression, and community identity are negotiated and performed. Anguelovski [2] emphasizes how neighborhoods and public spaces function as sites of refuge, reconstruction, and environmental justice, particularly in diverse urban contexts. Similarly, Low, Taplin, and Scheld [32] conceptualize urban parks as socially constructed public spaces where cultural diversity becomes visible and negotiated through everyday practices. More recently, Monaco [37] highlights the territorial and identity dimensions of sustainability, arguing that place-based identities shape how sustainability goals are perceived and enacted. Engaging with these perspectives allows us to situate our three-dimensional framework within broader debates on urban green spaces as spaces of social meaning, identity formation, and cultural negotiation.

### Case, data and methods

According to the Swiss Labour Force Survey (SLFS) [45], as of 2021, the permanent resident population with a migration background in Switzerland accounted for 39% (2,890,000 individuals). The majority, approximately four-fifths, belonged to the first generation (2,276,000), while the remaining fifth consisted of individuals born in Switzerland, making up the second generation (615,000). According to the Federal Statistical Office, Zurich, the most populous city in Switzerland, has the highest number of foreign residents in absolute terms, totaling 124,823 individuals. As of 2020, non-Swiss residents comprise 32.2% of Zurich’s total population, with the majority (80%) originating from European countries [24]. Among Zurich’s total population, 46.9% were born abroad. Within the foreign population, 69.7% come from European Union (EU) or European Free Trade Association (EFTA) member states, while 30.3% originate from non-EU/EFTA countries (SLFS 2021). This diversity makes Switzerland and the city of Zurich an ideal setting to examine how cultural backgrounds influence the perceptions, preferences, and utilization patterns of UGAs [5, 6, 41]. More specifically, Zurich represents a meaningful case for examining culturally differentiated park use for three reasons. First, the city combines a high proportion of foreign-born residents with a well-established tradition of integrating social and ecological considerations into public green space planning [17,41]. This makes it possible to examine how culturally diverse populations engage with relatively well-developed urban green infrastructure. Second, Zurich reflects broader European urban trends of increasing cultural diversification within a stable welfare and governance regime, allowing insights that may be transferable to other European metropolitan contexts. Third, public parks in Zurich vary considerably in size, vegetation structure, and socio-spatial context, enabling an exploration of how cultural background interacts with different park typologies within the same institutional setting. For this study, urban green areas with diverse characteristics were selected: UP1 – Platzspitz, UP2 – Bäckeranlage, UP3 – Irchelpark, and UP4 – Rieterpark. These parks differ in size, location (central versus central-peripheral), and vegetation structure (basic versus complex structures) (see Appendix 1), allowing for the exploration of how cultural background interacts with different types of urban green areas.

### Go along interviews

Walking or go-along interviews were introduced as a qualitative research technique two decades ago [29]. Initially employed by ethnographers and human geographers, this method involved accompanying individuals, either on foot or in a vehicle, while conducting interviews to expand the scope of fieldwork and explore socio-spatial relationships [1]. Since then, walking or go-along interviews have become an essential component of the broader “mobility turn” in social sciences, emphasizing the significance of movement, including walking [43]. Consequently, this approach has gained popularity as a method of data collection across diverse research disciplines, including urban studies and social sciences. The walking/go-along interview technique was chosen because it enables the collection of context-sensitive insights into individuals’ interactions with others and with their surrounding environment.

A semi-structured interview guide was developed to ensure that key topics were addressed while maintaining flexibility for participants to express their perspectives in depth. The guide addressed key themes such as cultural background, frequency and purpose of park visits, perceived benefits of the UGAs, and suggestions for improvement. Open-ended questions were used to encourage participants to share personal stories, experiences, and perspectives, eliciting rich, detailed responses that provide a deeper understanding of how different users interact with and perceive parks. All interviews were recorded with participants’ consent and subsequently transcribed for detailed qualitative analysis. Moreover, participants signed a consent form, authorizing the use of data for research purposes, and were informed that all information would be treated confidentially and anonymously. The Ethics Committee of the Faculty of Business, Economics, and Social Sciences at the University of Bern has approved this research project.

These interviews were conducted by the first and fourth authors of this article, one with a Western European cultural background and the other with a non-Western European background. This approach allowed to benefit from the interviewers’ diverse cultural perspectives, foster openness in participants’ responses, and cover several languages spoken in Zurich. Still, it is acknowledged that interviewer positionality may have influenced interaction dynamics and participants’ openness, particularly in cases where cultural or linguistic backgrounds overlapped. The walking or go-along interviews took place in the four designated parks between March and April 2024, from 10:00 AM to 6:00 PM. A total of 100 interviews were conducted, 25 in each park, during eight field visits held on both weekdays and weekends, in the mornings and afternoons. Interviews lasted between 6 and 20 min. While moving through each park, users were systematically approached by selecting approximately every fifth person. Approximately one third of those approached agreed to participate. The interviews followed a survey questionnaire (see Appendix 3), resulting in quantifiable answers to questions (yes or no, or choice of categories), as well as additional stories and explanations spontaneously told by interview partners or solicitated by the interviewers. This results in both quantitative and qualitative pieces of information from each interview.

During our interviews, several contextual factors influenced the process and outcomes (see Appendix 1 and 2). Offering chocolate proved highly effective as an incentive, significantly boosting engagement with potential interviewees. However, it was nearly impossible to interview individuals who were eating, engaged in sports, or part of a group, suggesting a potential sampling bias. Families and dog owners emerged as the most approachable participants for interviews, likely due to their more relaxed presence in the parks. Favorable weather conditions also contributed to more casual and unhurried interviews, resulting in elaborate, sometimes overly detailed responses. In parks in more affluent areas, people showed greater receptivity to our interviews, understood questions easily, and engaged more willingly in conversation. These various factors collectively shape both the dynamics and quality of our collected data.

The walking/go-along method offers several strengths. Conducting interviews while moving through the park allows participants to respond to environmental stimuli in real time, generating situated and context-sensitive reflections. It also facilitates more spontaneous and embodied narratives compared to stationary interviews. However, this method also has limitations. The mobile format can lead to shorter responses, distractions, or environmental interruptions (e.g., noise, weather). It may also disadvantage individuals engaged in specific activities (e.g., sports, group gatherings), potentially contributing to sampling bias. These methodological characteristics should be considered when interpreting the findings.

### Operationalizing cultural background

Studies on ethnicity and outdoor recreation have employed various approaches to differentiate between ethnic affiliations in European contexts. Some studies have focused on the most common or largest immigrant groups in the country or city under investigation [3, 10, 26, 38, 39], while others have utilized census data to classify ethnic groups [13, 21]. Another approach involves categorizing immigrants based on their length of stay in the host country [42]. However, these three methods of classifying participants’ ethnic backgrounds present certain challenges. One issue is that the broad classifications often overlook intra-ethnic variations, treating ethnic minorities as homogenous groups without acknowledging internal diversity. These groupings are primarily based on statistical factors (such as the most common minority in the country or census categorization). In some cases, ethnic minority status is assigned based on citizenship, failing to capture cultural nuances. Moreover, citizenship status does not necessarily indicate the level of acculturation. To address limitations of conventional ethnic classification approaches, walking/go-along interviews were used to gain deeper insights into participants’ cultural backgrounds, including indirect questions that allowed for a more nuanced understanding of self-identification.

A Swiss cultural background was defined as applying to individuals born in Switzerland who speak at least one of the Swiss national languages as their native language. However, for participants who reported a Swiss national language as their mother tongue, additional questions were asked to verify the classification. The interview responses allowed us to get a deeper understanding of participants’ cultural background beyond language. For analytical purposes, park users were divided into two groups: the Swiss group (born in Switzerland with a Swiss national language as mother tongue) and the non-Swiss group (all others). The non-Swiss group includes both migrants to Switzerland and tourists visiting the country. Given the exploratory design and sample size, a parsimonious grouping strategy was adopted, and the internal heterogeneity of the non-Swiss category is acknowledged as a limitation.

Our dataset includes participants from 26 different non-Swiss nationalities, with distribution between Swiss and non-Swiss individuals at 51% and 49%, respectively (see Appendix 4 for a discussion of alternative approach to classification). In our sample, female participants outnumbered male users in both groups. In the Swiss category, 56.9% of the participants were female, and 43.1% were male, while in the non-Swiss category, 57.1% were female, and 42.9% were male. The average age was 47 in the Swiss category compared to 39 in the non-Swiss category. While not perfectly balanced, the gender distribution is comparable across both categories.

## Results

According to the framework proposed above, the study distinguishes three main dimensions of individuals’ park use and preferences. First, the motivational dimension includes types of park use and individuals’ motivation for visiting the park. Second, the emotional dimension covers satisfaction with the park and emotions experienced during park visits. Third, the ecological dimension captures the importance of nature for park users and the role of vegetation structure in parks.

### Motivational dimension

**Fig. 1:**
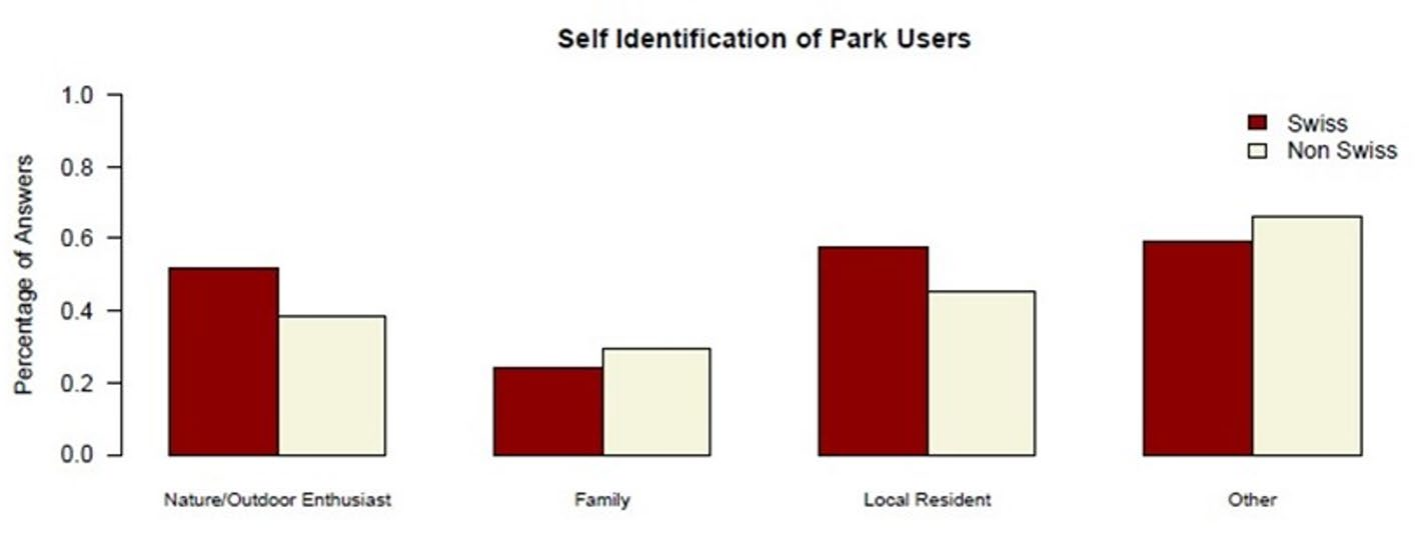
Self-identification of park users by cultural background. *N* = 100 (Swiss *n* = 51;non-Swiss *n* = 49). Cultural background was classified based on country of birth and native language (see Methods). Percentages refer to respondents within each cultural group. Where multiple responses were possible, percentages may exceed 100%

The motivational dimension is crucial for understanding how and why people use parks and thus provides insights into the diverse reasons that attract different user groups (Fig. 1).

Differences between Swiss and non-Swiss users were observed particularly in three categories: “Nature/Outdoor Enthusiast,” “Family,” and “Local Resident.”. As shown in Fig. 1, the “Nature/Outdoor Enthusiast” category was more prevalent among Swiss users, with 51.9% identifying with this category, compared to 38.6% among non-Swiss users. Additionally, 57.4% of Swiss participants and 45.5% of non-Swiss participants identified as “Local Residents”. The Family category was more commonly chosen by non-Swiss users (29.5%) than Swiss users (24.1%).

“Nature/Outdoor Enthusiasts” category is more likely prevalent among Swiss users than non-Swiss participants. This may indicate a stronger inclination towards nature-related activities within the Swiss users. One Swiss participant, describing their park habits, commented, *“I am a jogger and walker*,* an occasional user. I prefer being fully in nature*,* where I can also be active. But during the transitional seasons*,* it’s nice to have a park nearby for lunch.”* (Interview 70, female, 45). This statement highlights the dual function of parks, as both convenient urban retreats and spaces for those seeking deeper connections with nature. Another Swiss user appreciated the beauty and functionality of parks, noting, *“It’s a beautiful park. I like parks and it’s near her home. And she said it’s a very nice place. I like that it has free places to sit and picnic and also benches if you want to sit on a bench and also the view is beautiful.”* (Interview 79, female, 20). This observation may indicate a balance between aesthetic pleasure and practical amenities that parks offer to their users.

The “Family” category appears to be more prevalent among non-Swiss users. A non-Swiss user noted, *“Here you can recover and meditate. I prefer to come here on weekends in the summer to do yoga*,* to run*,* it’s very important; and walking there is a small hill too*,* it’s important; all three of us walk*,* the whole family with the child.”* (Interview 63, female, 31). This may demonstrate how parks are valued as spaces for both physical activity and family bonding for non-Swiss users. An observation of a non-Swiss user that is related to family activities noted that *“People come with children*,* and kindergartens walk with children too*,* well*,* and I think students who just run sometimes do sports; many people run.”* (Interview 66, female, 51). This may suggest that the park accommodates a diverse range of users, including families and students.Users who selected the “Local Resident” category explained this choice as a form of self-identification, indicating proximity to the parks regardless of their cultural background. One non-Swiss resident mentioned, *“I live about 20 minutes from here. I also like culture as there are a few events here around Christmas. There are some festivals.”* (Interview 1, male, 70). This reflects the dual appeal of the park for both leisure and cultural engagement. A Swiss visitor shared, “*I live near the Hartplatz. That’s where I usually find my green spaces. It’s difficult for me to perceive the life of marginalized people as it is here. If I were here with a child*,* I would make sure they don’t go near the group because it’s not a sight for a child. But my green spaces are more family-friendly.*” (Interview 31, female, 62). Another non-Swiss individual remarked, “*I have a dog*,* but I usually don’t go here with her. I have children*,* so it’s quite convenient with children.*” (Interview 39, female, 50). These statements are consistent with concerns about the park’s environment while also acknowledging its convenience for family activities.

The second aspect of the motivational dimension is assessed as the individual motivation to visit the park, with participants able to select multiple motivations.

**Fig. 2:**
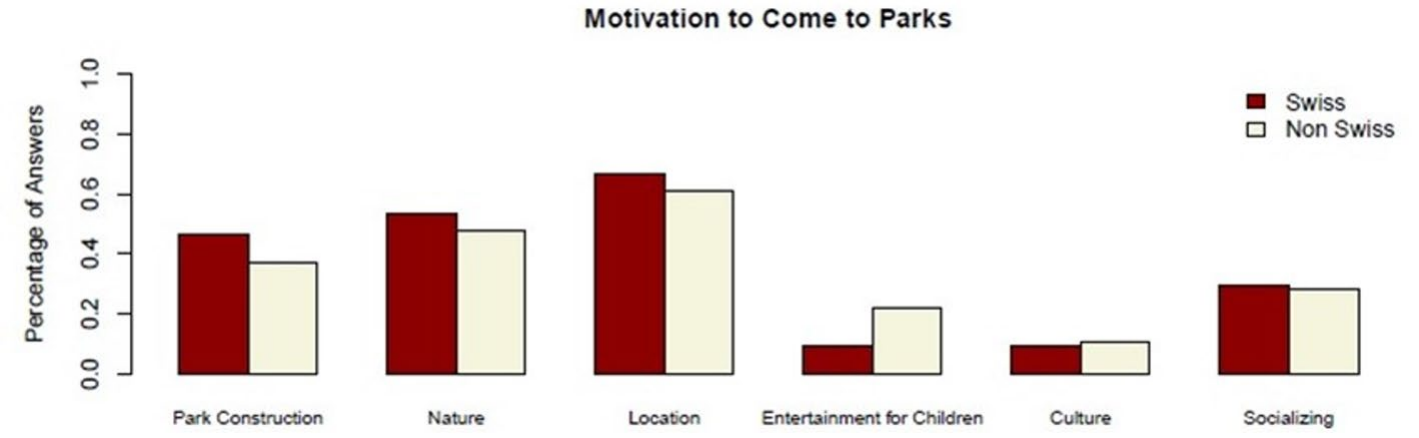
Motivations for park visits by cultural background. *N* = 100 (Swiss *n* = 51; non-Swiss *n* = 49). Cultural background was classified based on country of birth and native language (see Methods). Percentages refer to respondents within each cultural group. Where multiple responses were possible, percentages may exceed 100%

As shown in Fig. 2, “Park Construction,” “Location,” and “Nature” were the most commonly selected motivations across both groups. These aspects were reported more frequently by participants categorized as Swiss (46.3%, 53.7%, and 66.7%, respectively) than by those categorized as non-Swiss (37%, 47.8%, and 60.9%). For non-Swiss users, “Entertainment for Children” and “Culture” (21.7%, 10.9%) are more important compared to Swiss users (9.3%, 9.3%). Socializing is almost equally important for both groups (29.6% for Swiss users versus 28.3% for non-Swiss users).

The response “Park Construction” refers to the aesthetic value and maintenance quality of parks. Swiss respondents were more inclined to highlight the importance of well-maintained parks, with one stating: *“It is close*,* centrally located*,* and beautiful. It has a bit of nature. It’s not all concrete.”* (Interview 8, female, 34). Another Swiss participant emphasized the aesthetic value, mentioning: *“The new annex of the Landesmuseum. The architecture. Time to enjoy the sun.”* (Interview 9, male, 59).

Swiss participants more frequently cited “Nature” as a motivation to visit a park. One explained, *“Nature*,* the greenery. It’s a bit quieter here than in traffic. The possibility to relax brings me here.”* (Interview 11, male, 45). Another Swiss visitor appreciated the beauty of the park, stating, *“It’s very great*,* especially now when the flowers are here and when the trees come.”* Participants also valued the convenient location of parks. As one non-Swiss noted, *“It’s close to the train station. So while we’re waiting*,* it’s nice to have someplace quiet.”* (Interview 19, male, 26). The combination of nature and social interactions was significant too, as one non-Swiss visitor shared, *“It has an open area*,* nature*,* and the people here are calming.”* (Interview 32, male, 26).

For non-Swiss participants, children’s entertainment was more frequently identified as an important motivation compared to Swiss participants, as illustrated by the following statement, *“My granddaughter lives here. We come because of her*,* so she can experience some nature*,* and the air is also better.”* (Interview 6, female, 67). Another non-Swiss visitor appreciated parks for outdoor activities, with one noting: *“I come here to see all the family and the children. We have picnics together.”* (Interview 1, male, 70).

### Emotional dimension

The emotional dimension focuses on satisfaction with the park and emotions experienced during park visits. The analysis indicates that, overall, non-Swiss participants reported higher levels of satisfaction with parks in Zurich than Swiss users.Non-Swiss users were generally more satisfied with parks with comments such as *“Switzerland is the best quality”* (Interview 12, male, 43), *“Saint Petersburg where I am from*,* despite having many beautiful parks*,* is not as green as here*,* so I am very satisfied.”* (Interview 63, female, 31). Non-Swiss respondents often compared these areas with parks from their home countries, noting that Swiss parks are “greener” and better maintained.

In contrast, several Swiss participants expressed more critical reflections on what they consider to be *“*nature.*”* For example, one Swiss user stated: *“Nature is not electricity*,* no cars*,* no noise—animals*,* birds.”* (Interview 20, male, 34). Another respondent emphasized seclusion and reduced traffic noise: *“Seclusion. Even if it’s only on a small scale here. Little traffic noise. This might actually be the wrong place for that. But in the middle of the park it might be better*.*”* (Interview 29, male, 40). Similarly, a Swiss participant remarked: *“Nature has to do with calmness and animals. With life*,* with greenery*,* with plants. But when I say I go into nature*,* I don’t mean this park. Basically*,* there is a lot of greenery and nature here*,* and it is well maintained*,* which is important. But if you ask me what it means to go into nature*,* it’s more about being away from noise and from people.”* (Interview 43, male, 42).

These statements may suggest that while non-Swiss participants often expressed appreciation for infrastructure and the aesthetic qualities of urban green areas, Swiss participants in our sample tended to articulate more demanding or idealized expectations of what “nature” should entail.

The emotional dimension includes the feelings of users in parks. Many interviewees reported feeling relaxed or calm, prompting the researchers to add an additional category, “Calmness,” to capture this sentiment.

**Fig. 3:**
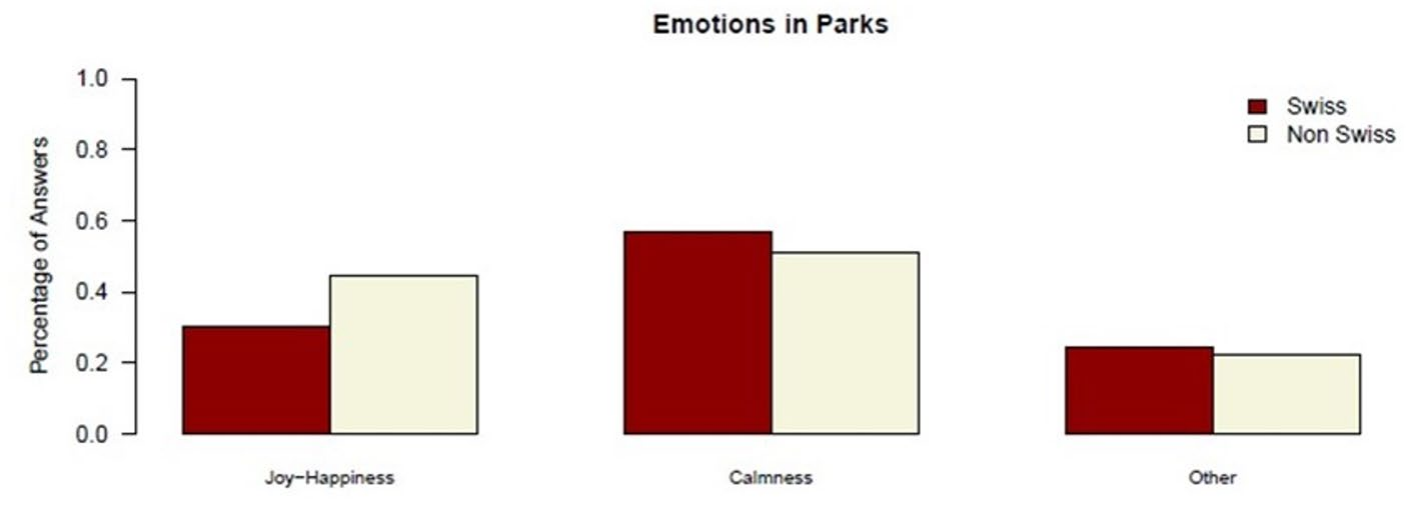
Emotions for park visits by cultural background. *N* = 100 (Swiss *n* = 51; non-Swiss *n* = 49). Cultural background was classified based on country of birth and native language (see Methods). Percentages refer to respondents within each cultural group. Where multiple responses were possible, percentages may exceed 100%

Figure 3 shows the distribution of emotions according to different cultural groups. It reveals that “Joy-Happiness” is most frequently reported by non-Swiss participants (44.4% vs. 30.2% for the Swiss participants). In contrast, “Calmness” is an emotion which is prominent among the Swiss group (56.6% vs. 51.1% for the non-Swiss group). The “Other” category includes feelings such as fear or surprise.

A non-Swiss visitor described the park as evoking a feeling of serenity, *“It’s serenity. We feel good. We feel at peace.”* (Interview 84, female, 62). This is also reflected in responses of another non-Swiss user such as *“I feel relaxed and calm”* (Interview 56, female, 40). On the other hand, Swiss users also emphasized the feeling of joy or happiness stating for example: “*Nostalgia. I am happy. It’s joy*,* happy.*” (Interview 15, male, 54).

However, fear is also a recurring emotion in these parks, particularly due to concerns over safety and the presence of drug users. One Swiss participant noted, “*Security is important for the older people. It’s good that the police are here every day to get the “Drogendealer” away from this park.*” (Interview 1, male, 70). This statement reflects concerns about safety in centrally located urban parks, which may experience higher foot traffic and a broader mix of users.The analysis highlights distinct emotional patterns across the two groups. Non-Swiss participants in this sample more frequently associated parks with joy and happiness, whereas Swiss participants more often emphasized calmness and serenity.

### Ecological dimension

**Fig. 4:**
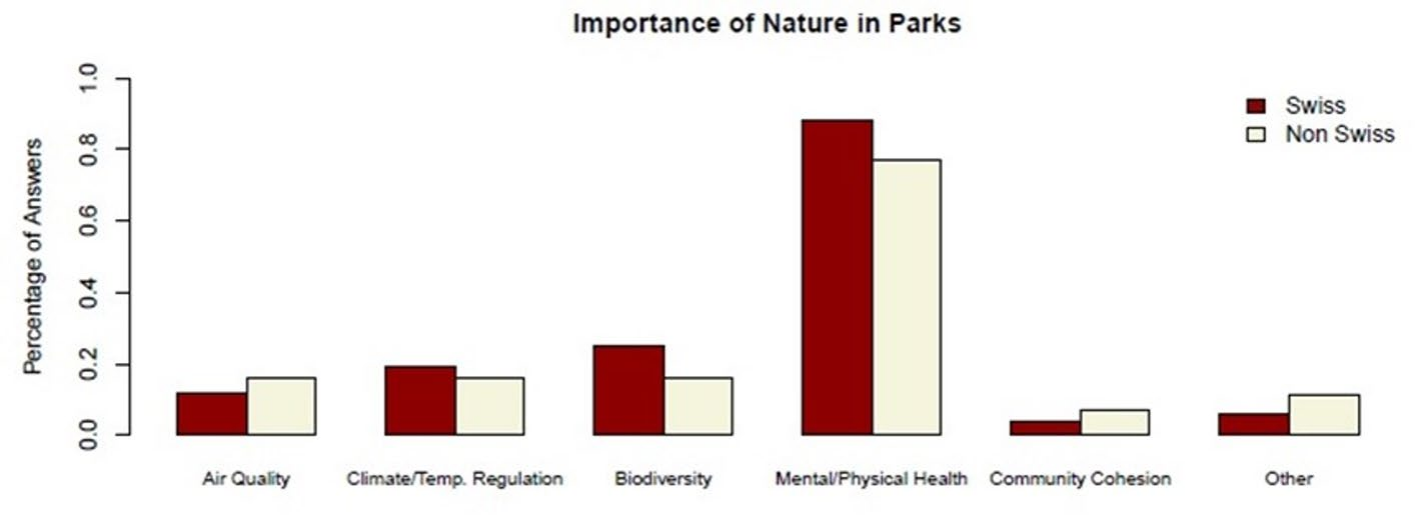
Reasons of importance of nature in parks by cultural background. *N* = 100 (Swiss *n* = 51; non-Swiss *n* = 49). Cultural background was classified based on country of birth and native language (see Methods). Percentages refer to respondents within each cultural group. Where multiple responses were possible, percentages may exceed 100%

The ecological dimension captures the importance of nature for park users and the role of vegetation structure in parks. Most respondents emphasized the importance of nature in parks, primarily for its mental and physical health benefits, making it the most frequently mentioned reason for valuing nature in parks (88.5% for Swiss respondents vs. 77.3% for non-Swiss users), as shown in Fig. 4. This was followed by “Biodiversity” (25.0% for Swiss vs. 15.9% for non-Swiss users), “Climate/Temperature Regulation” (19.2% for Swiss vs. 15.9% for non-Swiss users), and “Air Quality” (11.5% for Swiss vs. 15.9% for non-Swiss users).

As mentioned, many respondents emphasized “Mental and Physical Health” as the primary reason for valuing nature in parks. One non-Swiss respondent highlighted the restorative aspect of parks: *“Here you can recover and meditate. I prefer to come here on weekends in the summer to do yoga*,* to run*,* it’s very important. Walking here is great; there’s a small hill*,* and all three of us walk together as a family with our child.”* (Interview 63, female, 31). A non-Swiss participant linked parks to urban well-being: *“For me*,* a park is something inherently connected to nature. For those who live in big cities*,* it is important to have a place to clear their minds and recharge.”* (Interview 64, female, 36). Nature’s presence in cities was also noted as a key factor for a non-Swiss user: *“I think nature is important in every city and every park. When I see a city with many parks*,* I just enjoy it. I’ve been to Barcelona*,* and there weren’t many parks—that was annoying for me. But here*,* I see nature*,* I see parks. It’s very good.”* (Interview 18, female, 21).

As shown in Fig. 4, Swiss users in this sample placed greater emphasis on “Biodiversity” and “Climate/Temperature Regulation” than the non-Swiss group. This pattern may suggest differences in how nature-related functions are prioritized, rather than indicating a stronger emotional connection per se. One Swiss respondent expressed appreciation for urban biodiversity, stating, *“The fact that there is biodiversity in the city is great. It doesn’t necessarily have to be in the park. Even along the streets. If parking spaces were removed and replaced with biodiverse flower beds*,* that would be very good.”* (Interview 45, male, 79). Another Swiss respondent highlighted the importance of nature for both animals and people, remarking, *“In general*,* for the animals*,* for everything. Also*,* for the people. It is important. It is beautiful. I like it. You shouldn’t always trample on it*,* otherwise it will take revenge.”* (Interview 55, female, 74). Another Swiss participant noted the broader implications of biodiversity, saying, *“It still provides a habitat for creatures and humans to coexist. You know it is safe if you compare it to other places where nature is dangerous… The topic of biodiversity is one of the key global threats that we have identified.”* (Interview 95, male, 46).

In contrast, the non-Swiss group placed greater emphasis on “Community Cohesion” as an important aspect of nature in parks, suggesting these individuals might use these spaces differently, particularly for more social interactions. A non-Swiss user emphasized the social benefits of parks, stating, *“To be able to breathe. To be able to rest. To be able to recharge. To be able to meet with friends. People are happy. That’s important.”* (Interview 84, female, 62). Similarly, a non-Swiss user underscored park’s role as a community hub, saying, *“The park is important for the neighborhood as a meeting place. It’s not just about streets and concrete but also about having a place to meet.”* (Interview 100, male, 39).

The second aspect of the ecological dimension assesses preferences for vegetation structure in parks: Minimally, moderately, or highly structured vegetation. Swiss users have a slight preference for “Highly Structured Vegetation” compared to non-Swiss users (37.5% vs. 36.8%). “Moderately Structured Vegetation” is slightly more preferred among non-Swiss users (62.1% versus 57.7%). “Minimally Structured Vegetation” received too few responses to be meaningfully included in our analysis.

The participants’ explanations reveal a range of different perspectives on vegetation structure in parks. Many participants highlighted the importance of trees. For instance, a Swiss respondent commented, *“The tree with flowers is beautiful”* (Interview 1, male, 70). Another Swiss respondent expressed the important function of trees, saying, *“The tall trees are great*,* especially for shade.”* (Interview 87, female, 43). The same function of trees is also identified by other Swiss users, with one stating: “*Shade in the summer is important”* (Interview 5, male, 74). Another aspect mentioned by various users is the need for vegetation diversity in parks. A non-Swiss respondent said: “*I always like diversity in a park. But we choose to sit here*,* so we like a lot of nature. Not wild nature*,* but a lot of it”* (Interview 19, male, 26). A Swiss participant echoed similar sentiments, saying, *“The mix is important. The tall trees are great*,* especially for shade. The mix is beautiful. Tall trees are very important. It shouldn’t just be lawns. We really appreciate the trees and bushes”* (Interview 87, female, 43). Participants expressed diverse views on the balance between nature and infrastructure. One non-Swiss respondent focused on a mix between the two, stating: *“The mix is important*,* including between infrastructure and nature”* (Interview 7, male, 39). While a non-Swiss respondent apparently preferred more infrastructure noting; *“There isn’t a lot of infrastructure*,* but I take what I get here”* (Interview 10, male, 30). Additionally, varied preferences on landscape design were mentioned. Some participants expressed their love for well-maintained nature and open spaces with a non-Swiss participant mentioning: *“I always like trees. It’s better without the scrub*,* because where I lived before*,* they had trees people could sit under. This is better without the scrub”* (Interview 14, male, 34). Mostly Swiss respondents voiced their preferences for wilder nature, with one stating: *“a little bit wild. The wildest one. It’s nature. We live in a city*,* and that’s why nature is important”* (Interview 82, female, 80). *This may reflect a stronger connection to nature for Swiss people.*

## Discussion and conclusions

This article analyzed whether park users with different cultural backgrounds perceive and use parks differently. For the analysis, a three-dimensional framework—motivational, emotional, and ecological—was introduced to investigate park use and to provide structured insights into the relationship between cultural background and engagement with urban green areas (UGAs). By examining the interplay of cultural diversity and ecological characteristics of UGAs, this study builds on previous work [12, 41] to provide implications for inclusive urban green space planning. Our insights address inclusivity and urban resilience in the context of growing, diversifying cities, in times when UGAs are deemed increasingly important also for biodiversity or climate change adaptation. The research contributes to knowledge on UGAs use and perception within the European context, where such studies remain relatively scarce [14, 19].

Results reveal both shared perceptions and distinct preferences among park users of different cultural backgrounds. Across both groups, parks are highly valued for their contribution to mental and physical health, citing this as the primary benefit. Additionally, location and proximity to nature are consistently prioritized as key factors motivating park visits, suggesting that accessibility is crucial for park usage. However, the study also highlights cultural differences in park use and perception.

The motivational dimension is crucial for understanding how and why people use parks, as it provides insights into the diverse reasons that attract different user groups. Park use motivations appear to vary across cultural backgrounds. For example, users categorized as Swiss tend to place greater emphasis on aesthetics and outdoor activities, whereas non-Swiss users are more likely to highlight children’s entertainment and park infrastructure, suggesting a more family-oriented approach to park visits. In terms of self-identification, Swiss respondents in the sample were more inclined to identify as nature and outdoor enthusiasts, while participants categorized as non-Swiss more frequently referred to family-related uses. These findings align with previous research on park use and perception in multicultural contexts, such as Seeland et al. [42] and Dai [14]. The emphasis on family orientation among non-Swiss users is also consistent with Gentin’s [19] findings, which identified infrastructure as a key motivator for park visits among family-oriented groups in urban settings.

The emotional dimension of park use is important because it captures how individuals psychologically engage with public green spaces, thereby shaping their reported well-being and overall experience. General satisfaction, as well as specific emotions such as joy, calmness, or fear, influences how these spaces are perceived and experienced. A more detailed understanding of these aspects can inform urban planning discussions aimed at fostering inclusive and supportive environments.

In the present sample, satisfaction levels vary across groups. Swiss participants reported higher satisfaction in parks characterized by more natural features (UP3 and UP4), whereas participants categorized as non-Swiss expressed more heterogeneous views regarding parks with more artificial elements (UP1 and UP2). These patterns may suggest that Swiss users in this sample tend to favor less developed, more natural environments, while non-Swiss users appear to value infrastructural amenities in urban parks.

Emotional responses also differ descriptively between groups. Joy and happiness were more frequently reported among non-Swiss participants, particularly in parks with greater infrastructure, possibly reflecting the importance of social gatherings and shared activities in these settings. These reported patterns are consistent with Gobster [20], who highlights the role of social interaction in shaping emotional experiences in parks. Swiss participants more frequently mentioned feelings of surprise, which may relate to the appreciation of unexpected natural elements or encounters within park environments. This observation resonates with Peters’ [38] discussion of the restorative effects of unanticipated natural features. By contrast, some non-Swiss participants reported feelings of fear, which may reflect differing perceptions of safety and comfort in urban green spaces. Considering emotional responses such as joy or calmness contributes to a broader understanding of well-being in natural environments, as discussed by Chiesura [12], while the present study adds a culturally differentiated perspective to this discussion.

The ecological dimension further illustrates how park characteristics are perceived. Urban nature is widely associated in the literature with benefits such as mental and physical health, biodiversity support, improved air quality, and climate regulation. Preferences regarding vegetation structure also vary descriptively across groups. In this sample, Swiss participants expressed a stronger preference for more structured environments (e.g., lawns combined with large trees and shrubs), whereas non-Swiss participants more often favored simpler vegetation structures. These differences echo prior discussions on culturally shaped aesthetic preferences [26, 41]. The preference for structured environments among Swiss respondents is broadly consistent with Peters [38], while the emphasis on simplicity among non-Swiss users may relate to accessibility or functional considerations, as suggested by Gentin [19].

Finally, the perceived importance of nature in parks also differs descriptively: Swiss participants more frequently emphasized biodiversity and climate regulation, whereas non-Swiss participants more often highlighted community cohesion, describing parks as spaces for social interaction and connection.

This study has several limitations. First, the study faced several challenges that potentially impacted the diversity of perspectives captured through the go-along interview approach which may have introduced sampling bias. At UP1, a self-selection bias emerged, as participants were primarily individuals who were alone or with small children, excluding those who were eating, engaged in sports, or part of a group. Interview timing and weather conditions also impacted participant availability and willingness to engage, resulting in fewer and less detailed responses during Monday morning interviews at UP2 or under bad weather conditions at UP3. Additionally, the physical and social environments of the parks influenced the data collection. UP2, a smaller park with a mixed-use environment, presented sampling challenges due to its polarized spaces – one side dominated by homeless individuals and drug users, the other side by families. The presence of construction noise further deterred participants from engaging in extended interviews, potentially reducing response depth. At UP3, the sample skewed towards a more educated and culturally diverse demographic, which may have led to an overrepresentation of certain perspectives. Similarly, UP4 predominantly affluent user base largely excludes marginalized groups, in contrast to the more diverse composition at UP2. While the study did not systematically analyze other individual-level variables, such as age or gender (e.g. [12, 27, 41, 42]),, it aimed for a balanced distribution across the parks. Although age or gender might potentially influence preferences, there is no evidence to suggest that they interact distinctively with cultural background concerning park use. Data collection was conducted between March and April. Seasonal conditions may influence park use, vegetation perception, and emotional responses. Park visitation patterns, motivations, and experiences could differ during summer, winter, or peak tourist seasons. Therefore, the findings should be interpreted as reflecting a specific seasonal context. Finally, it is important to note that cultural differences transcend the simplified distinction between Swiss and non-Swiss groups, and other variables (e.g., socioeconomic status or ethnicity) likely shape park users’ experiences and preferences. The operationalization of cultural background as a dichotomous variable does not distinguish between long-term migrants and temporary visitors, nor does it account for other influential factors such as socioeconomic status, ethnicity, age, or gender. This categorization enabled an exploratory comparison of broad cultural backgrounds based on the assumption that longer-term socialization processes regarding use and perception of UGA differ between Swiss and non-Swiss individuals, as suggested by research on culturally shaped nature preferences and migrant experiences in urban forests [10, 26]. Yet, the simple dichotomy does not capture the internal heterogeneity within these groups. Future research could move beyond the Swiss/non-Swiss dichotomy by differentiating between migration histories, length of residence, generational status, and socio-economic positioning. Such differentiation would allow for a more nuanced understanding of how cultural identity, integration processes, and social stratification intersect in shaping park use and perception. Longitudinal designs could further examine how preferences and emotional attachments evolve over time, particularly among migrants and second-generation residents. Comparative studies across cities or governance regimes would also help assess the transferability of the three-dimensional framework and determine whether similar patterns emerge in urban contexts with different planning traditions, welfare systems, or integration policies. Expanding this approach to other metropolitan regions would therefore contribute to a broader understanding of culturally differentiated park use within diverse institutional and socio-political settings.

While the quantitative analysis remains descriptive and percentages are used to illustrate patterns across groups, no statistical tests were conducted to assess the significance of these differences; the findings should therefore be interpreted as indicative patterns rather than statistically validated group differences. Future research could expand in both directions: toward more robust statistical inference (with a larger number of observations and clearer identification of key variables), or toward deeper qualitative analysis (with fewer observations but greater attention to causal narratives linking multiple variables).

Overall, the results emphasize the importance of addressing cultural diversity and inclusivity in UGAs, as advocated by Seeland et al. [42] and UN Habitat [47]. By considering both shared and distinct cultural preferences, urban planners and policymakers can better align with the call for inclusivity and sustainability in cities [43]. Balancing natural and aesthetic elements with social infrastructure can enhance the appeal of parks and fulfill the diverse needs of urban populations, as called for by Chiesura [12] and Gentin [19].

These findings support broader perspectives that conceptualize parks as socially constructed spaces of belonging and negotiation (Low et al. [32]) and as sites where environmental justice and recognition unfold (Anguelovski [2]). The differentiated emphasis on biodiversity versus community cohesion further aligns with Monaco’s [37] argument that sustainability is shaped by place-based identities. This approach contributes directly to the implementation of UN Agenda 2030 strategies, ensuring UGAs are inclusive and resilient.

## Data Availability

The data will be stored as Eawag data (WP2 and WP3-4 data will be stored at WSL). It is Eawag’s policy to generally retain all relevant data generated or used in research projects in Eawag’s institutional research data repository.

## Appendix

**Appendix 1.** Main characteristics of the case studies – urban parks Platzspitz (UP1), Bäckeranlage (UP2), Irchelpark (UP3) and Rieterpark (UP4)

**Table:**

Location	Historical Significance	Social Dynamics	Socio-demographic Context	Design and Infrastructure	Greening and Biodiversity	Main Users	Size
UP1	Central, between Sihl and Limmat rivers, surrounded by business area.	Baroque architecture, used as hunting grounds in the Middle Ages.	Family-friendly, diverse visitors, historical site with Tibetan community gatherings.	Privileged area, blend of Baroque and modern design, pathways, and recreational facilities.	Landscaped greenery, moderate biodiversity.	Local residents, tourists, nature, and cultural enthusiasts.	6.5 ha
UP2	Periphery of the center, surrounded by residential area.	Alemannic cemetery turned bourgeois park in 1901, renovated in 1939.	Mixed social activities, from family gatherings to youth recreation.	Less privileged area, family-friendly design, pathways, and café.	Mixed greenery, moderate biodiversity.	Local residents, families, workers, cultural, and nature enthusiasts.	1.5 ha
UP3	Periphery of the center, near university and residential area.	Established in the late 20th century as part of the University of Zurich campus.	Popular among university students, staff, and local residents.	Privileged area, modern design, open spaces, and sports facilities.	Extensive greenery, moderate biodiversity.	Students, academic staff, residents, researchers, and nature enthusiasts.	36 ha
UP4	Periphery of the center, surrounded by residential areas.	One of Zurich’s oldest public parks, dating back to the 19th century.	Communal space for leisure, family gatherings, and cultural events.	Privileged area, combines natural and historical elements with modern amenities.	Landscaped greenery, contributes to urban biodiversity.	Local residents, Rietberg Museum visitors, tourists, joggers, dog owners, nature, art and culture enthusiasts.	26 ha

The information in the table is based on information from the city of Zurich (official website), field visits, park observations, and survey results. In the survey, we asked users how they identify themselves as park users and, in their opinion, who the main users of the park are. This helped us identify the primary user groups for each park. These findings were complemented with information provided by the City of Zurich to ensure that all relevant categories of main park users were adequately represented.

**Appendix 2.** Table summarizing the key observations and interview details from the park surveys.

**Table:**

Park	Key Observations	Interview Conduct
UP1	Morning interviews were difficult due to fewer people. More potential interviewees later in the day. Struggled with culturally diverse and younger people.	Conducting interviews while walking led to more open responses. The method requires experience for accuracy. Challenges with specific questions.
UP2	Inviting spring weather; diverse user groups (families, dog owners, substance users, workers). Noise from construction impacted interviews. Challenging to approach due to park’s small size and mixed weather.	Interviews were tricky due to noise and understanding of questions. Diverse user groups coexisted peacefully despite park small size.
UP3	Larger park with complex plant structures and biodiversity info. Mostly students, dog owners, families. No tourists or marginalized people.	Bad weather made finding participants difficult and led to shorter interviews during first survey day. Students understood and responded well to the survey during second day.
UP4	Relaxed and positive atmosphere, mostly well-off visitors. Families, dog owners, joggers, museum visitors. No marginalized people.	Easy to approach well-educated visitors. Longer interviews due to relaxed Saturday mood. High understanding of survey.

**Appendix 3.** Survey questions.

**Table:**

№	Survey Question	Response Options
1	**How regularly do you visit this park?**	Spontaneously/on fixed days
2	***Motivation of individuals to visit the park/cultural ecosystem services*** **What motivates you to come to the park? (Multiple answers possible)**	Aesthetics/Park design/Park maintenance/Nature/Location/Park infrastructure/Events/Entertainment for children/Attractions/Interaction - exchange with others/Outdoor gathering with family and friends/Picnic/barbeque/Providing services-selling-maintenance/History-heritage-statues/Sport/Others (please specify)
3	***Satisfaction with the park*** **Are you satisfied with the current state of this park?**	Very satisfied/Satisfied/Neutral/Not Satisfied/I don’t know
4	**What wishes for change do you have?**	Open-ended
5	***Preferred structural complexity of the park*** **Which area in the park do you prefer? Why?**	
6	***Types of park use*** **How do you identify yourself as a user of this park? (Multiple answers possible)**	Local Resident/Tourist/Family/Worker/Jogger and Walker/Dog owner/Cyclist/Nature Enthusiast/Outdoor Enthusiast/Cultural Enthusiast/University Student/Academic Staff/Researchers/Others (please specify)
7	**In your experience**,** who are the main users of this park (e.g.**,** cultural communities**,** age groups)?**	Open-ended
8	**Do you think the park is equally attractive for different users: younger and older generations**,** culturally diverse people**,** and those with physical disabilities?**	Yes/No
9	***Emotions experienced when visiting the park*** **What kind of emotions do you have in this park? (multiple answers possible)**	Joy-happiness/Trust/Fear/Surprise/Sadness/Disgust/Anger/Anticipation/Other (please specify)
10	**Does the park hold any special significance for you: Memories? Nostalgia? Historical Importance?**	Yes/No
11	**What does the term “nature” mean for you?**	Open-ended
12	***Importance of nature for park users*** **Do you think nature is important in this park? Why? (Multiple answers possible)**	Air Quality/Climate/Temperature Regulation/Biodiversity/Water Management/Mental and Physical Health/Community Cohesion/Economic Value/All of them/None of them/Other
13	**How do you define your gender?**	Male/Female/Rather not say/Other
14	**What is your age?**	Open-ended
15	**What is your country of birth?**	Open-ended
16	**What is your native language?**	Open-ended
17	**Do you regularly watch and read Swiss news?**	Yes/No
18	**Are you first or second-generation living in Switzerland?**	First/Second/None of them
19	**Did/do you attend a Swiss educational institution?**	Yes/No
20	**How strongly do you identify yourself as Swiss?**	Very strongly/Strongly/Little bit/Not at all

**Appendix 4. Cultural background**,** gender and average age distribution in the survey**.

Cultural background was additionally assessed through related survey questions in order to compare and validate the initial classification. To the question “Are you first or second-generation living in Switzerland?“, 54% of respondents identified as Swiss, while 46% identified as non-Swiss. To the question “How do you identify yourself as Swiss?“, 57% Swiss individuals chose the “very strongly” and “strongly” response categories, whereas 43% chose “a little bit” and “not at all” response categories. All 51 identified Swiss users (100%) confirmed that they have attended or are currently attending a Swiss educational institution. In contrast, only 19 of the identified non-Swiss users (38.78%) out of 49 indicated that they have studied or are studying at a Swiss educational institution.

**Appendix 5. Socio-demographic Characteristics of Selected Interview Participants**

**Table:**

Interview No.	Park name	Date of interview	How do you define your gender?	What is your age?	What is your country of birth?	What is your native language?	Cultural Background
1	Platzspitz	11.03.2024	Male	70	Tibet	Tibetan	Non-Swiss
5	Platzspitz	11.03.2024	Male	74	Switzerland	Swiss German	Swiss
6	Platzspitz	11.03.2024	Female	67	Tibet	Swiss German	Non-Swiss
7	Platzspitz	11.03.2024	Male	39	Germany	German, French	Non-Swiss
8	Platzspitz	11.03.2024	Female	34	Switzerland	Swiss German	Swiss
9	Platzspitz	11.03.2024	Male	59	Switzerland	Swiss German	Swiss
10	Platzspitz	11.03.2024	Male	30	China	Chinese	Non-Swiss
11	Platzspitz	11.03.2024	Male	45	Switzerland	Swiss German	Swiss
12	Platzspitz	11.03.2024	Male	43	Turkey	Turkish	Non-Swiss
14	Platzspitz	16.03.2024	Male	34	India	Bengali	Non-Swiss
15	Platzspitz	16.03.2024	Male	54	Switzerland	Swiss German	Swiss
18	Platzspitz	16.03.2024	Female	21	Ukraine	Ukrainian	Non-Swiss
19	Platzspitz	16.03.2024	Male	26	Denmark	Danish	Non-Swiss
20	Platzspitz	16.03.2024	Male	34	Switzerland	Swiss German	Swiss
29	Bäckeranlage	16.03.2024	Male	40	Switzerland	Swiss German	Swiss
31	Bäckeranlage	16.03.2024	Female	62	Switzerland	Swiss German	Swiss
32	Bäckeranlage	16.03.2024	Male	26	France	French	Non-Swiss
39	Bäckeranlage	25.03.2024	Female	50	United Kingdom	English	Non-Swiss
43	Bäckeranlage	25.03.2024	Male	42	Switzerland	Swiss German	Swiss
45	Bäckeranlage	25.03.2024	Male	79	Switzerland	Swiss German	Swiss
55	Irchelpark	17.03.2024	Female	74	Switzerland	Swiss German	Swiss
56	Irchelpark	17.03.2024	Female	40	Ukraine	Ukrainian	Non-Swiss
63	Irchelpark	08.04.2024	Female	31	Russia	Russian	Non-Swiss
64	Irchelpark	08.04.2024	Female	36	Azerbaijan	Russian	Non-Swiss
66	Irchelpark	08.04.2024	Female	51	Germany	Ukrainian	Non-Swiss
70	Irchelpark	08.04.2024	Female	45	Switzerland	Swiss German	Swiss
79	Rieterpark	13.04.2024	Female	20	Switzerland	Swiss German	Swiss
82	Rieterpark	13.04.2024	Female	80	Switzerland	Swiss German	Swiss
84	Rieterpark	13.04.2024	Female	62	France	French	Non-Swiss
87	Rieterpark	13.04.2024	Female	43	Switzerland	Swiss German	Swiss
95	Rieterpark	10.05.2024	Male	46	Switzerland	Swiss German	Swiss
100	Rieterpark	10.05.2024	Male	39	Slovakia	Slovak	Non-Swiss

Table presents selected sociodemographic characteristics of interviewed participants. Cultural categorization follows the operationalization described in the Methods section.
